# Phenyl acridine-9-carboxyl­ate

**DOI:** 10.1107/S1600536813002055

**Published:** 2013-01-31

**Authors:** Michał Wera, Damian Trzybiński, Karol Krzymiński, Jerzy Błażejowski

**Affiliations:** a Faculty of Chemistry, University of Gdańsk, J. Sobieskiego 18, 80-952 Gdańsk, Poland

## Abstract

The acridine ring system and the benzene ring in the title compound, C_20_H_13_NO_2_, are oriented at a dihedral angle of 6.4 (2)°. The carboxyl group is twisted at an angle of 83.6 (2)° relative to the acridine skeleton. The mol­ecules in the crystal are arranged in stacks along the *b* axis, with two of the acridine rings involved in multiple π–π inter­actions [centroid–centroid distances in the range 3.536 (2)–3.894 (2) Å]. Stacks arranged parallel are linked *via* C—H⋯π inter­actions, forming layers in the *ac* plane that are in contact with adjacent, inversely oriented layers *via* other C—H⋯π inter­actions, giving rise to double layers. The inversely oriented double layers inter­act dispersively. The acridine units are parallel within the parallel-oriented stacks, but inclined at an angle of 79.6 (2)° in the inversely oriented stacks.

## Related literature

For general background to the applications of the title compound, see: Krzymiński *et al.* (2011 ▶); Natrajan *et al.* (2012 ▶); Trzybiński *et al.* (2010 ▶). For related structures, see: Trzybiński *et al.* (2013 ▶). For inter­molecular inter­actions, see: Hunter *et al.* (2001 ▶); Takahashi *et al.* (2001 ▶). For the synthesis, see: Sato (1996 ▶); Trzybiński *et al.* (2010 ▶).
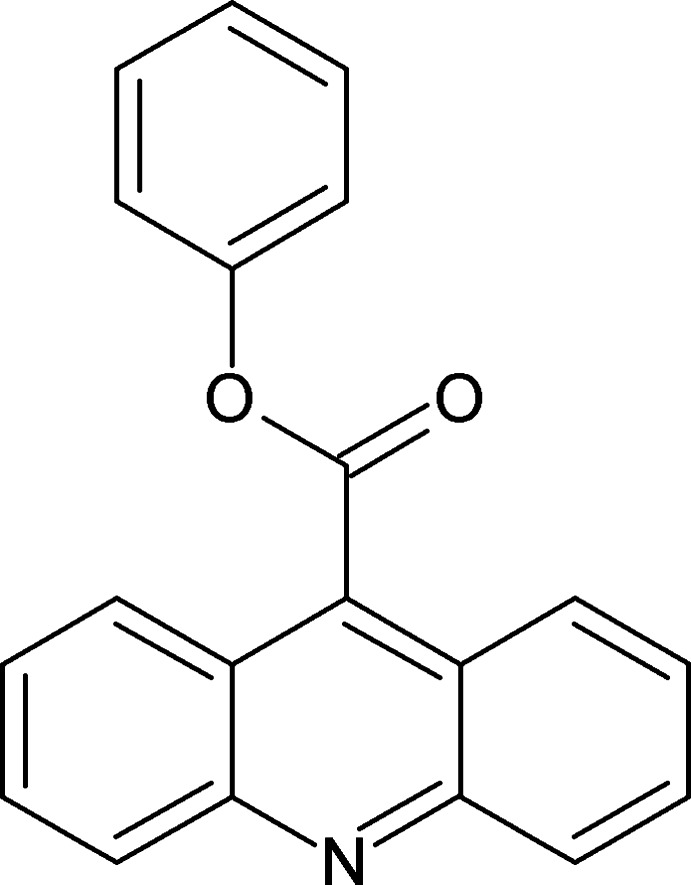


## Experimental

### 

#### Crystal data

C_20_H_13_NO_2_
*M* *_r_* = 299.31
Monoclinic, 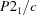

*a* = 17.094 (2) Å
*b* = 5.4175 (7) Å
*c* = 16.310 (2) Å
β = 95.545 (11)°
*V* = 1503.3 (3) Å^3^
*Z* = 4
Mo *K*α radiation
μ = 0.09 mm^−1^
*T* = 295 K
0.6 × 0.2 × 0.1 mm

#### Data collection

Oxford Diffraction Gemini R Ultra Ruby CCD diffractometer
Absorption correction: multi-scan (*CrysAlis RED*; Oxford Diffraction, 2008 ▶) *T*_min_ = 0.354, *T*_max_ = 0.986
9221 measured reflections
2651 independent reflections
1560 reflections with *I* > 2σ(*I*)
*R*_int_ = 0.068

#### Refinement

*R*[*F* ^2^ > 2σ(*F* ^2^)] = 0.073
*wR*(*F* ^2^) = 0.203
*S* = 1.04
2651 reflections
209 parameters
H-atom parameters constrained
Δρ_max_ = 0.29 e Å^−3^
Δρ_min_ = −0.34 e Å^−3^

### 

Data collection: *CrysAlis CCD* (Oxford Diffraction, 2008 ▶); cell refinement: *CrysAlis RED* (Oxford Diffraction, 2008 ▶); data reduction: *CrysAlis RED*; program(s) used to solve structure: *SHELXS97* (Sheldrick, 2008 ▶); program(s) used to refine structure: *SHELXL97* (Sheldrick, 2008 ▶); molecular graphics: *ORTEP-3* (Farrugia, 2012 ▶); software used to prepare material for publication: *SHELXL97* and *PLATON* (Spek, 2009 ▶).

## Supplementary Material

Crystal structure: contains datablock(s) global, I. DOI: 10.1107/S1600536813002055/xu5671sup1.cif

Structure factors: contains datablock(s) I. DOI: 10.1107/S1600536813002055/xu5671Isup2.hkl

Supplementary material file. DOI: 10.1107/S1600536813002055/xu5671Isup3.cml

Additional supplementary materials:  crystallographic information; 3D view; checkCIF report

## Figures and Tables

**Table 1:** Hydrogen-bond geometry (Å, °) *Cg*2 and *Cg*4 denote the centroids of the C1–C4/C11/C12 and C18–C23 rings, respectively.

*D*—H⋯*A*	*D*—H	H⋯*A*	*D*⋯*A*	*D*—H⋯*A*
C3—H3⋯*Cg*2^i^	0.93	2.98	3.712 (3)	137
C7—H7⋯*Cg*4^ii^	0.93	2.84	3.646 (3)	145

Symmetry codes: (i) 
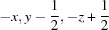
; (ii) 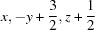.

**Fig. 1.**
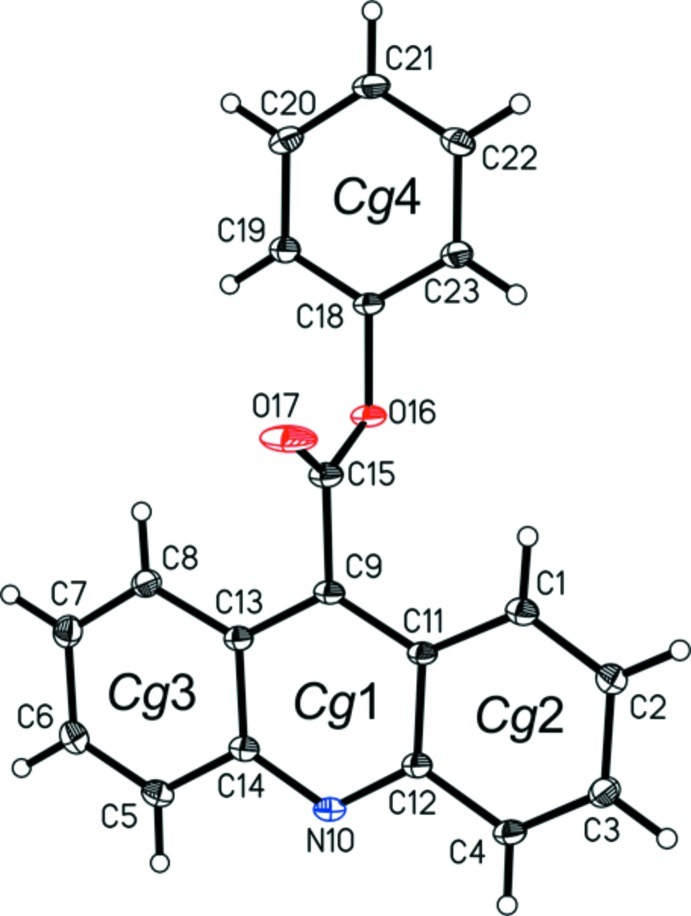
The molecular structure of the title compound showing the atom-labeling scheme. Displacement ellipsoids are drawn at the 25% probability level and H atoms are shown as small spheres of arbitrary radius. Cg1, Cg2, Cg3 and Cg4 denote the ring centroids.

**Fig. 2.**
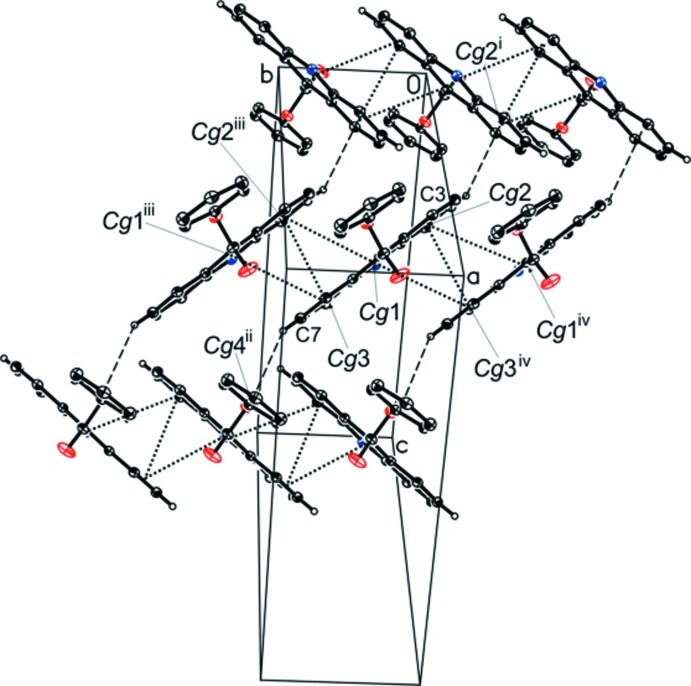
The arrangement of the molecules in the crystal structure. The C–H···π interactions are represented by dashed lines, the π–π contacts by dotted lines. H atoms not involved in the interactions have been omitted. [Symmetry codes: (i) –x, y – 1/2, –z + 1/2; (ii) x, –y + 3/2, z + 1/2; (iii) x, y + 1, z; (iv) x, y – 1, z.]

**Fig. 3.**
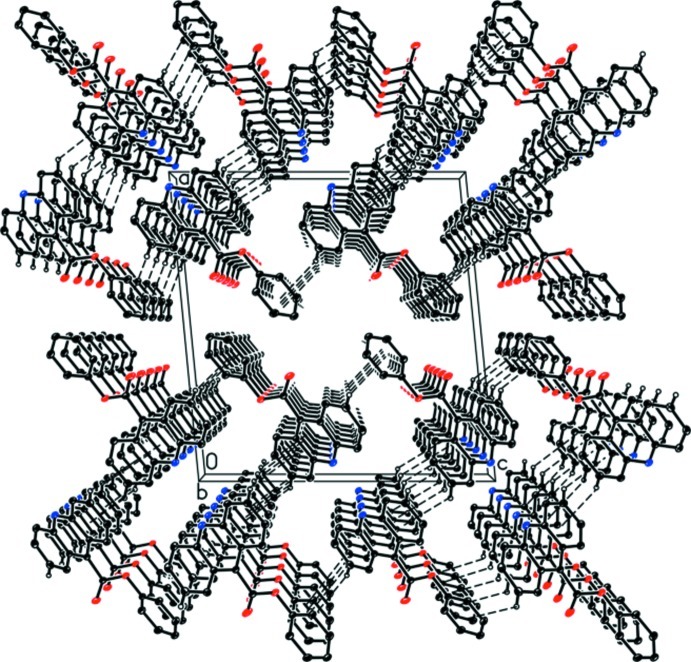
Molecular stacks in the crystal structure, viewed along the b axis. The C–H···π interactions are represented by dashed lines. H atoms not involved in the interactions have been omitted.

## supplementary crystallographic information

### Comment

Phenyl acridine-9-carboxylates are the precursors of 9-(phenoxycarbonyl)-10-methylacridinium salts, whose cations exhibit a chemiluminogenic ability that can be utilized analytically (Natrajan *et al.*, 2012). Here we present the structure of phenyl acridine-9-carboxylate, the precursor of a basic chemiluminogen in this group of compounds, whose structure (Trzybiński *et al.*, 2010) and chemiluminogenic features (Krzymiński *et al.*, 2011) have recently been investigated.

The bond lengths and angles characterizing the geometry of the acridine and phenyl moieties of the title compound (Fig. 1) are similar to those found in phenyl acridine-9-carboxylates alkyl-substituted at the benzene ring, investigated earlier (Trzybiński *et al.*, 2013, and references cited therein). With respective average deviations from planarity of 0.0143 (3) Å and 0.0037 (3) Å, the acridine ring system and the benzene ring are oriented at a dihedral angle of 6.4 (2)° [this angle varies between 30.0 (2)° – 37.7 (1)°, as indicated by the data for phenyl acridine-9-carboxylates alkyl-substituted at the benzene ring, investigated earlier (Trzybiński *et al.*, 2013, and the references cited therein)]. The carboxyl group is twisted at an angle of 83.6 (2)° relative to the acridine skeleton [this angle varies between 58.0 (2)° – 68.1 (2)° as indicated by the data for phenyl acridine-9-carboxylates alkyl-substituted at the benzene ring, investigated earlier (Trzybiński *et al.*, 2013, and the references cited therein)].

The search for intermolecular interactions in the crystal using PLATON (Spek, 2009) has shown that the parallel oriented molecules of the title compound (Fig. 2) are arranged in stacks along the *b* axis (Fig. 3) in which two of the acridine rings are involved in multiple π–π interactions (Table 2, Fig. 2) of an attractive nature (Hunter *et al.*, 2001). The stacks arranged parallel linked via C–H···π interactions, of an attractive nature (Takahashi *et al.*, 2001), form layers in the *ac* plane that are in contact with adjacent, inversely-oriented such layers via other C–H···π interactons giving rise to double layers (Table 1, Figs. 2 and 3). The inversely oriented double layers interact dispersively. The acridine moieties are parallel within the stacks oriented in parallel, but inclined at an angle of 79.6 (2)° in the inversely oriented stacks. This interesting crystal architecture to some extent resembles the crystal structure of 2,6-dimethylphenyl acridine-9-carboxylate (Trzybiński *et al.*, 2013).

### Experimental

Phenyl acridine-9-carboxylate was synthesized by the esterification of 9-(chlorocarbonyl)acridine (obtained in the reaction of acridine-9-carboxylic acid with a tenfold molar excess of thionyl chloride) with phenol in anhydrous dichloromethane in the presence of N,N-diethylethanamine and a catalytic amount of N,N-dimethyl-4-pyridinamine (room temperature, 15 h) (Sato, 1996; Trzybiński *et al.*, 2010). The product was purified chromatographically (SiO_2_, cyclohexane/ethyl acetate, 3/2 v/v). Pale-yellow crystals suitable for X-ray investigations were grown from cyclohexane (m.p. 463–464 K).

### Refinement

H atoms were positioned geometrically, with C–H = 0.93 Å, and constrained to ride on their parent atoms with *U*_iso_(H) = 1.2*U*_eq_(C).

### Crystal data

**Table:**

C_20_H_13_NO_2_	*F*(000) = 624
*M**_r_* = 299.31	*D*_x_ = 1.322 Mg m^−^^3^
Monoclinic, *P*2_1_/*c*	Mo *K*α radiation, λ = 0.71073 Å
Hall symbol: -P 2ybc	Cell parameters from 2651 reflections
*a* = 17.094 (2) Å	θ = 3.3–25.0°
*b* = 5.4175 (7) Å	µ = 0.09 mm^−^^1^
*c* = 16.310 (2) Å	*T* = 295 K
β = 95.545 (11)°	Needle, pale-yellow
*V* = 1503.3 (3) Å^3^	0.6 × 0.2 × 0.1 mm
*Z* = 4	

### Data collection

**Table:**

Oxford Diffraction Gemini R Ultra Ruby CCD diffractometer	2651 independent reflections
Radiation source: Enhanced (Mo) X-ray Source	1560 reflections with *I* > 2σ(*I*)
Graphite monochromator	*R*_int_ = 0.068
Detector resolution: 10.4002 pixels mm^-1^	θ_max_ = 25.0°, θ_min_ = 3.3°
ω scans	*h* = −17→20
Absorption correction: multi-scan (*CrysAlis RED*; Oxford Diffraction, 2008)	*k* = −5→6
*T*_min_ = 0.354, *T*_max_ = 0.986	*l* = −16→19
9221 measured reflections	

### Refinement

**Table:**

Refinement on *F*^2^	Primary atom site location: structure-invariant direct methods
Least-squares matrix: full	Secondary atom site location: difference Fourier map
*R*[*F*^2^ > 2σ(*F*^2^)] = 0.073	Hydrogen site location: inferred from neighbouring sites
*wR*(*F*^2^) = 0.203	H-atom parameters constrained
*S* = 1.04	*w* = 1/[σ^2^(*F*_o_^2^) + (0.1073*P*)^2^] where *P* = (*F*_o_^2^ + 2*F*_c_^2^)/3
2651 reflections	(Δ/σ)_max_ < 0.001
209 parameters	Δρ_max_ = 0.29 e Å^−^^3^
0 restraints	Δρ_min_ = −0.34 e Å^−^^3^

### Special details

**Table:**

Geometry. All esds (except the esd in the dihedral angle between two l.s. planes) are estimated using the full covariance matrix. The cell esds are taken into account individually in the estimation of esds in distances, angles and torsion angles; correlations between esds in cell parameters are only used when they are defined by crystal symmetry. An approximate (isotropic) treatment of cell esds is used for estimating esds involving l.s. planes.
Refinement. Refinement of F^2^ against ALL reflections. The weighted R-factor wR and goodness of fit S are based on F^2^, conventional R-factors R are based on F, with F set to zero for negative F^2^. The threshold expression of F^2^ > 2sigma(F^2^) is used only for calculating R-factors(gt) etc. and is not relevant to the choice of reflections for refinement. R-factors based on F^2^ are statistically about twice as large as those based on F, and R- factors based on ALL data will be even larger.

### Fractional atomic coordinates and isotropic or equivalent isotropic displacement parameters (Å^2^)

**Table:**

	*x*	*y*	*z*	*U*_iso_*/*U*_eq_	
C1	0.15883 (16)	−0.0807 (5)	0.28634 (16)	0.0564 (7)	
H1	0.2005	−0.0842	0.2537	0.068*	
C2	0.09977 (17)	−0.2461 (5)	0.27301 (17)	0.0602 (8)	
H2	0.1012	−0.3623	0.2312	0.072*	
C3	0.03597 (17)	−0.2442 (5)	0.32180 (18)	0.0622 (8)	
H3	−0.0042	−0.3592	0.3118	0.075*	
C4	0.03260 (16)	−0.0781 (5)	0.38254 (17)	0.0573 (7)	
H4	−0.0098	−0.0801	0.4142	0.069*	
C5	0.13817 (18)	0.5964 (5)	0.54258 (17)	0.0621 (8)	
H5	0.0945	0.5900	0.5724	0.075*	
C6	0.1938 (2)	0.7688 (5)	0.56161 (18)	0.0690 (8)	
H6	0.1877	0.8807	0.6038	0.083*	
C7	0.26132 (19)	0.7811 (5)	0.51795 (19)	0.0673 (8)	
H7	0.2996	0.9000	0.5318	0.081*	
C8	0.27029 (17)	0.6208 (5)	0.45642 (17)	0.0581 (7)	
H8	0.3149	0.6312	0.4281	0.070*	
C9	0.21784 (14)	0.2683 (4)	0.36980 (15)	0.0473 (7)	
N10	0.08713 (13)	0.2595 (4)	0.46103 (13)	0.0548 (6)	
C11	0.15783 (14)	0.0982 (4)	0.34973 (15)	0.0451 (6)	
C12	0.09280 (15)	0.1001 (4)	0.39897 (15)	0.0489 (7)	
C13	0.21349 (14)	0.4373 (4)	0.43382 (15)	0.0479 (7)	
C14	0.14481 (15)	0.4254 (4)	0.47830 (15)	0.0506 (7)	
C15	0.29032 (16)	0.2631 (5)	0.32487 (17)	0.0552 (7)	
O16	0.28060 (10)	0.3857 (3)	0.25400 (11)	0.0581 (6)	
O17	0.34884 (13)	0.1610 (5)	0.34906 (14)	0.1104 (10)	
C18	0.34637 (15)	0.3952 (5)	0.20676 (15)	0.0486 (7)	
C19	0.39837 (17)	0.5859 (5)	0.21985 (17)	0.0603 (8)	
H19	0.3919	0.7045	0.2599	0.072*	
C20	0.46112 (17)	0.5984 (6)	0.1720 (2)	0.0695 (9)	
H20	0.4975	0.7259	0.1801	0.083*	
C21	0.46958 (17)	0.4238 (6)	0.11312 (19)	0.0695 (9)	
H21	0.5119	0.4321	0.0814	0.083*	
C22	0.41592 (19)	0.2364 (6)	0.10059 (19)	0.0699 (9)	
H22	0.4216	0.1193	0.0599	0.084*	
C23	0.35334 (17)	0.2201 (5)	0.14809 (18)	0.0613 (8)	
H23	0.3169	0.0927	0.1401	0.074*	

### Atomic displacement parameters (Å^2^)

**Table:**

	*U*^11^	*U*^22^	*U*^33^	*U*^12^	*U*^13^	*U*^23^
C1	0.0505 (18)	0.0670 (17)	0.0530 (16)	0.0065 (13)	0.0117 (13)	0.0049 (14)
C2	0.060 (2)	0.0634 (17)	0.0577 (17)	0.0004 (14)	0.0095 (14)	−0.0044 (14)
C3	0.0557 (19)	0.0651 (17)	0.0657 (19)	−0.0119 (13)	0.0046 (15)	0.0053 (16)
C4	0.0440 (17)	0.0697 (17)	0.0600 (17)	−0.0022 (13)	0.0151 (13)	0.0072 (15)
C5	0.065 (2)	0.0662 (17)	0.0579 (17)	0.0055 (15)	0.0196 (14)	−0.0019 (15)
C6	0.083 (2)	0.0668 (18)	0.0567 (18)	0.0026 (16)	0.0038 (16)	−0.0074 (15)
C7	0.071 (2)	0.0649 (18)	0.0643 (19)	−0.0087 (15)	−0.0035 (16)	0.0059 (16)
C8	0.0508 (18)	0.0667 (17)	0.0565 (17)	−0.0039 (13)	0.0045 (13)	0.0079 (14)
C9	0.0397 (15)	0.0578 (15)	0.0460 (14)	0.0069 (11)	0.0123 (11)	0.0092 (12)
N10	0.0488 (15)	0.0625 (13)	0.0561 (14)	−0.0013 (11)	0.0202 (11)	0.0040 (11)
C11	0.0348 (14)	0.0565 (14)	0.0453 (14)	0.0046 (11)	0.0111 (11)	0.0059 (13)
C12	0.0427 (16)	0.0568 (15)	0.0479 (15)	0.0019 (12)	0.0081 (12)	0.0084 (13)
C13	0.0415 (16)	0.0553 (15)	0.0477 (15)	0.0028 (11)	0.0077 (12)	0.0083 (12)
C14	0.0478 (17)	0.0557 (15)	0.0496 (15)	0.0049 (12)	0.0108 (12)	0.0071 (13)
C15	0.0421 (17)	0.0687 (17)	0.0564 (17)	0.0071 (13)	0.0132 (13)	0.0141 (14)
O16	0.0396 (11)	0.0788 (12)	0.0590 (11)	0.0076 (8)	0.0204 (8)	0.0163 (10)
O17	0.0564 (16)	0.182 (2)	0.0990 (18)	0.0486 (15)	0.0378 (13)	0.0715 (17)
C18	0.0376 (15)	0.0609 (15)	0.0493 (15)	0.0022 (12)	0.0140 (11)	0.0099 (13)
C19	0.0558 (18)	0.0657 (17)	0.0615 (17)	−0.0029 (14)	0.0169 (14)	−0.0034 (14)
C20	0.051 (2)	0.0773 (19)	0.082 (2)	−0.0141 (14)	0.0167 (16)	0.0079 (18)
C21	0.0485 (19)	0.096 (2)	0.0667 (19)	0.0011 (16)	0.0220 (15)	0.0150 (18)
C22	0.071 (2)	0.080 (2)	0.0629 (19)	0.0036 (16)	0.0253 (16)	−0.0085 (16)
C23	0.0560 (19)	0.0645 (17)	0.0662 (18)	−0.0090 (13)	0.0193 (14)	−0.0021 (15)

### Geometric parameters (Å, º)

**Table:**

C1—C2	1.352 (4)	C9—C15	1.500 (4)
C1—C11	1.418 (3)	N10—C12	1.341 (3)
C1—H1	0.9300	N10—C14	1.344 (3)
C2—C3	1.411 (4)	C11—C12	1.433 (3)
C2—H2	0.9300	C13—C14	1.440 (3)
C3—C4	1.343 (4)	C15—O17	1.177 (3)
C3—H3	0.9300	C15—O16	1.329 (3)
C4—C12	1.418 (4)	O16—C18	1.424 (3)
C4—H4	0.9300	C18—C23	1.361 (4)
C5—C6	1.347 (4)	C18—C19	1.366 (4)
C5—C14	1.412 (3)	C19—C20	1.387 (4)
C5—H5	0.9300	C19—H19	0.9300
C6—C7	1.415 (4)	C20—C21	1.366 (4)
C6—H6	0.9300	C20—H20	0.9300
C7—C8	1.347 (4)	C21—C22	1.370 (4)
C7—H7	0.9300	C21—H21	0.9300
C8—C13	1.413 (4)	C22—C23	1.383 (4)
C8—H8	0.9300	C22—H22	0.9300
C9—C11	1.394 (3)	C23—H23	0.9300
C9—C13	1.396 (3)		
			
C2—C1—C11	120.6 (3)	N10—C12—C4	118.5 (2)
C2—C1—H1	119.7	N10—C12—C11	123.0 (2)
C11—C1—H1	119.7	C4—C12—C11	118.5 (2)
C1—C2—C3	120.7 (3)	C9—C13—C8	124.9 (2)
C1—C2—H2	119.6	C9—C13—C14	116.9 (2)
C3—C2—H2	119.6	C8—C13—C14	118.2 (2)
C4—C3—C2	120.8 (3)	N10—C14—C5	119.0 (2)
C4—C3—H3	119.6	N10—C14—C13	122.8 (2)
C2—C3—H3	119.6	C5—C14—C13	118.2 (2)
C3—C4—C12	120.9 (3)	O17—C15—O16	123.8 (2)
C3—C4—H4	119.6	O17—C15—C9	124.1 (2)
C12—C4—H4	119.6	O16—C15—C9	112.1 (2)
C6—C5—C14	121.4 (3)	C15—O16—C18	116.72 (19)
C6—C5—H5	119.3	C23—C18—C19	122.5 (2)
C14—C5—H5	119.3	C23—C18—O16	118.9 (2)
C5—C6—C7	120.5 (3)	C19—C18—O16	118.6 (2)
C5—C6—H6	119.7	C18—C19—C20	118.4 (3)
C7—C6—H6	119.7	C18—C19—H19	120.8
C8—C7—C6	120.1 (3)	C20—C19—H19	120.8
C8—C7—H7	119.9	C21—C20—C19	120.1 (3)
C6—C7—H7	119.9	C21—C20—H20	119.9
C7—C8—C13	121.5 (3)	C19—C20—H20	119.9
C7—C8—H8	119.2	C20—C21—C22	120.2 (3)
C13—C8—H8	119.2	C20—C21—H21	119.9
C11—C9—C13	121.2 (2)	C22—C21—H21	119.9
C11—C9—C15	119.8 (2)	C21—C22—C23	120.4 (3)
C13—C9—C15	118.9 (2)	C21—C22—H22	119.8
C12—N10—C14	118.9 (2)	C23—C22—H22	119.8
C9—C11—C1	124.2 (2)	C18—C23—C22	118.3 (3)
C9—C11—C12	117.2 (2)	C18—C23—H23	120.8
C1—C11—C12	118.6 (2)	C22—C23—H23	120.8
			
C11—C1—C2—C3	0.0 (4)	C7—C8—C13—C14	0.2 (4)
C1—C2—C3—C4	0.0 (4)	C12—N10—C14—C5	178.8 (2)
C2—C3—C4—C12	0.3 (4)	C12—N10—C14—C13	−1.1 (4)
C14—C5—C6—C7	−0.8 (4)	C6—C5—C14—N10	−179.0 (3)
C5—C6—C7—C8	0.4 (4)	C6—C5—C14—C13	0.8 (4)
C6—C7—C8—C13	−0.2 (4)	C9—C13—C14—N10	0.8 (4)
C13—C9—C11—C1	−179.1 (2)	C8—C13—C14—N10	179.3 (2)
C15—C9—C11—C1	−2.1 (4)	C9—C13—C14—C5	−179.1 (2)
C13—C9—C11—C12	−1.7 (3)	C8—C13—C14—C5	−0.5 (3)
C15—C9—C11—C12	175.4 (2)	C11—C9—C15—O17	−95.7 (4)
C2—C1—C11—C9	177.2 (2)	C13—C9—C15—O17	81.4 (4)
C2—C1—C11—C12	−0.2 (4)	C11—C9—C15—O16	83.7 (3)
C14—N10—C12—C4	178.5 (2)	C13—C9—C15—O16	−99.2 (3)
C14—N10—C12—C11	−0.1 (3)	O17—C15—O16—C18	−1.4 (4)
C3—C4—C12—N10	−179.1 (2)	C9—C15—O16—C18	179.2 (2)
C3—C4—C12—C11	−0.5 (4)	C15—O16—C18—C23	92.5 (3)
C9—C11—C12—N10	1.4 (4)	C15—O16—C18—C19	−90.1 (3)
C1—C11—C12—N10	179.0 (2)	C23—C18—C19—C20	−0.9 (4)
C9—C11—C12—C4	−177.1 (2)	O16—C18—C19—C20	−178.2 (2)
C1—C11—C12—C4	0.4 (3)	C18—C19—C20—C21	0.5 (4)
C11—C9—C13—C8	−177.8 (2)	C19—C20—C21—C22	0.4 (4)
C15—C9—C13—C8	5.1 (4)	C20—C21—C22—C23	−0.9 (5)
C11—C9—C13—C14	0.6 (3)	C19—C18—C23—C22	0.5 (4)
C15—C9—C13—C14	−176.4 (2)	O16—C18—C23—C22	177.7 (2)
C7—C8—C13—C9	178.6 (2)	C21—C22—C23—C18	0.4 (4)

### Hydrogen-bond geometry (Å, º)

*Cg*2 and *Cg*4 denote the centroids of the C1–C4/C11/C12 and C18–C23 rings, respectively.

**Table:**

*D*—H···*A*	*D*—H	H···*A*	*D*···*A*	*D*—H···*A*
C3—H3···*Cg*2^i^	0.93	2.98	3.712 (3)	137
C7—H7···*Cg*4^ii^	0.93	2.84	3.646 (3)	145

Symmetry codes: (i) −*x*, *y*−1/2, −*z*+1/2; (ii) *x*, −*y*+3/2, *z*+1/2.

### π–π interactions (Å,°).

**Table:**

*I*	*J*	*CgI*···*CgJ*	Dihedral angle	*CgI*_Perp	Cg*I*_Offset
1	2^iii^	3.894 (2)	1.7 (2)	3.451 (1)	1.804 (1)
1	3^iv^	3.893 (2)	1.4 (2)	3.454 (1)	1.796 (1)
2	1^iv^	3.894 (2)	1.7 (2)	3.498 (2)	1.711 (2)
2	3^iv^	3.536 (2)	1.1 (2)	3.482 (2)	0.616 (2)
3	1^iii^	3.893 (2)	1.4 (2)	3.496 (2)	1.713 (2)
3	2^iii^	3.536 (2)	1.1 (2)	3.481 (2)	0.621 (2)

Symmetry codes: (iii) *x*, *y* + 1, *z*; (iv) *x*, *y* – 1, *z*.*Cg*1, *Cg*2 and *Cg*3 are the centroids of the C9/N10/C11–C14, C1–C4/C11/C12 and C5–C8/C13/C14 rings, respectively. Cg*I*···Cg*J* is the distance between ring centroids. The dihedral angle is that between the planes of the rings *I* and *J*. *CgI*_Perp is the perpendicular distance of *CgI* from ring *J*. Cg*I*_Offset is the distance between *CgI* and perpendicular projection of *CgJ* on ring *I*.
